# Impaired angiotensin II signaling in septic shock

**DOI:** 10.1186/s13613-024-01325-y

**Published:** 2024-06-14

**Authors:** Adrien Picod, Bruno Garcia, Dirk Van Lier, Peter Pickkers, Antoine Herpain, Alexandre Mebazaa, Feriel Azibani

**Affiliations:** 1 INSERM, UMR-S 942 MASCOT—Université Paris–Cité, Paris, France; 2 Department of Intensive Care Medicine, Centre Hospitalier Universitaire de Lille, Lille, France; 3 Experimental Laboratory of Intensive Care, Université Libre de Bruxelles, Brussels, Belgium; 4 Department of Intensive Care Medicine, Radboud University Medical Center, Nijmegen, The Netherlands; 5 Department of Intensive Care Medicine, St. Pierre University Hospital, Université Libre de Bruxelles, Brussels, Belgium; 6 Department of Anesthesiology, Burns and Critical Care, Hopitaux Saint-Louis—Lariboisière, AP-HP, Paris, France

**Keywords:** Renin–angiotensin–aldosterone system, Sepsis, Septic shock, Circulatory failure, Angiotensin II, Angiotensin-converting enzyme, Dipeptidyl peptidase 3, Neprilysin

## Abstract

Recent years have seen a resurgence of interest for the renin–angiotensin–aldosterone system in critically ill patients. Emerging data suggest that this vital homeostatic system, which plays a crucial role in maintaining systemic and renal hemodynamics during stressful conditions, is altered in septic shock, ultimately leading to impaired angiotensin II—angiotensin II type 1 receptor signaling. Indeed, available evidence from both experimental models and human studies indicates that alterations in the renin–angiotensin–aldosterone system during septic shock can occur at three distinct levels: 1. Impaired generation of angiotensin II, possibly attributable to defects in angiotensin-converting enzyme activity; 2. Enhanced degradation of angiotensin II by peptidases; and/or 3. Unavailability of angiotensin II type 1 receptor due to internalization or reduced synthesis. These alterations can occur either independently or in combination, ultimately leading to an uncoupling between the renin–angiotensin–aldosterone system input and downstream angiotensin II type 1 receptor signaling. It remains unclear whether exogenous angiotensin II infusion can adequately address all these mechanisms, and additional interventions may be required. These observations open a new avenue of research and offer the potential for novel therapeutic strategies to improve patient prognosis. In the near future, a deeper understanding of renin–angiotensin–aldosterone system alterations in septic shock should help to decipher patients’ phenotypes and to implement targeted interventions.

## Background

Septic shock represents the most severe form of sepsis. Septic shock is characterized by “profound circulatory, cellular, and metabolic abnormalities” and associated with high mortality [1]. Vasodilation is the primary feature of sepsis-associated circulatory failure, although a component of hypovolemia and/or myocardial depression is often present [2]. Accordingly, vasopressor support is the cornerstone of the symptomatic management of septic shock and relies on the use of norepinephrine on the frontline and vasopressin as a second line agent [3]. More recently, the use of angiotensin II has been suggested for catecholamine-refractory vasodilatory shock, mostly of septic origin [4]. The renin–angiotensin–aldosterone system (RAAS) is a complex homeostatic system with implication in numerous biological processes, with blood pressure maintenance and sodium homeostasis at the forefront. While overactivation of this system has been well-documented in chronic cardiovascular and kidney diseases, leading to a detrimental cycle of excessive vasoconstriction, sodium retention, and maladaptive repair, it is essential to remind that the RAAS primarily acts as a compensatory mechanism to cope with acute aggression. Accordingly, its integrity is crucial to maintain hemodynamics during circulatory stress. Recent data nevertheless suggest that this homeostatic mechanism is altered during the course of septic shock with potential therapeutic implications.

Starting with a comprehensive overview of the RAAS and its role in maintaining homeostasis, this concise translational review aims to summarize the available evidence regarding RAAS alterations during septic shock, and to discuss the consequences and potential therapeutic strategies.

### Overview of the RAAS

**Fig. 1:**
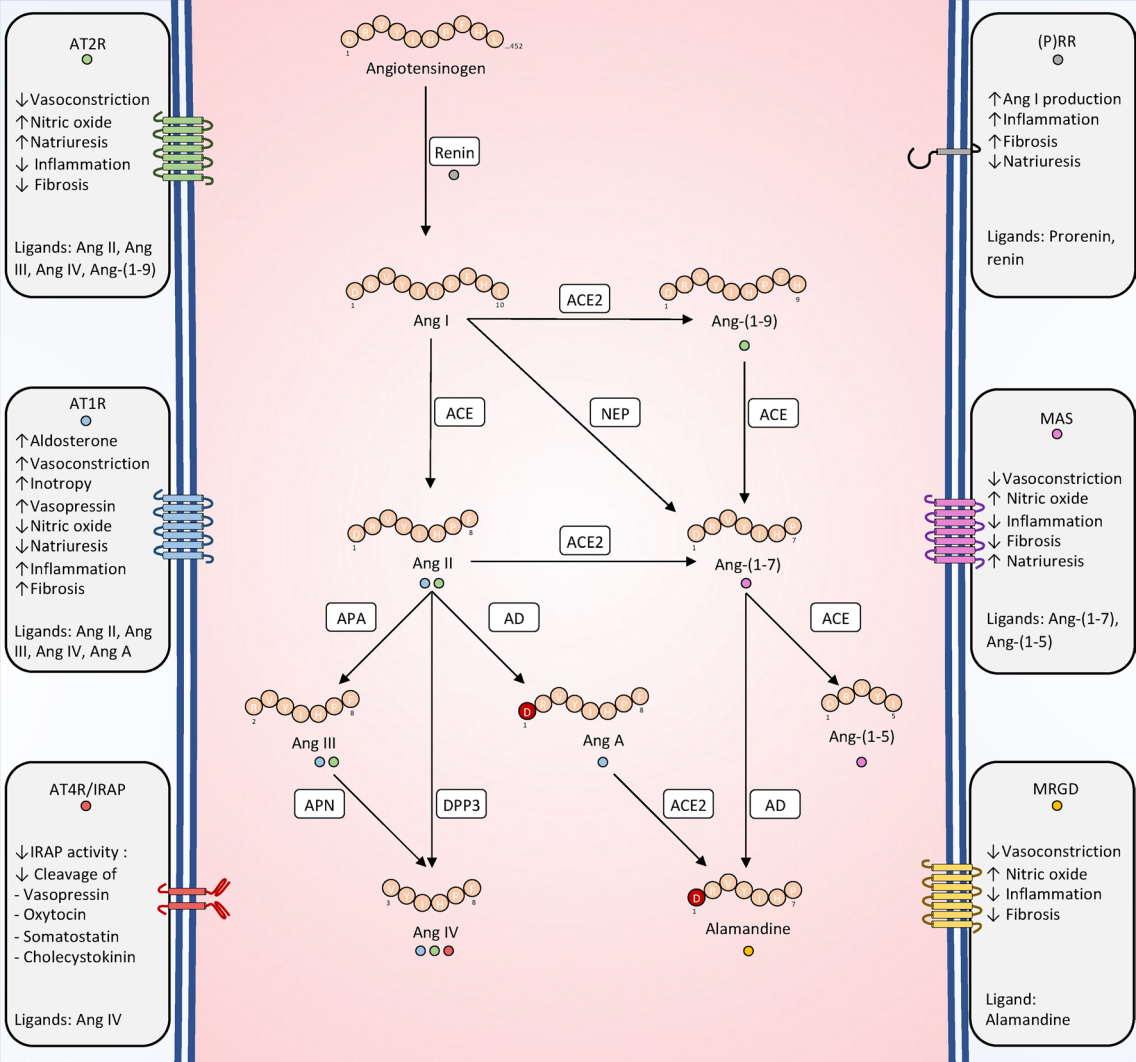
Overview of the RAAS. All angiotensin peptides originate from the sequential proteolytic cleavage of angiotensinogen. For clarity, only peptides with a firmly established biological function are depicted. The interactions of a given peptide with its receptor(s) are illustrated by the color-coded dot(s): blue (AT1R), green (AT2R), red (AT4R/IRAP), gray (PRR), purple (MAS) or yellow (MGRD). In addition to the ligand-receptor interactions depicted, a potential binding of Ang-(1–7) to AT1R at elevated concentrations has been reported. However, the biological significance of this interaction remains uncertain. *Ang* angiotensin, *ACE* angiotensin-converting enzyme, *ACE2* angiotensin-converting enzyme 2, *NEP* neprilysin, *DPP3* dipeptidyl peptidase 3, *APA* aminopeptidase A, *APN* aminopeptidase N, *AD* aspartate decarboxylase, *(P)RR* (pro-)renin receptor, *AT1R* angiotensin II, type 1 receptor, *AT2R* angiotensin II, type 2 receptor, *AT4R* angiotensin IV receptor; *MRGD* MAS-related G-protein-coupled D receptor

**Fig. 2:**
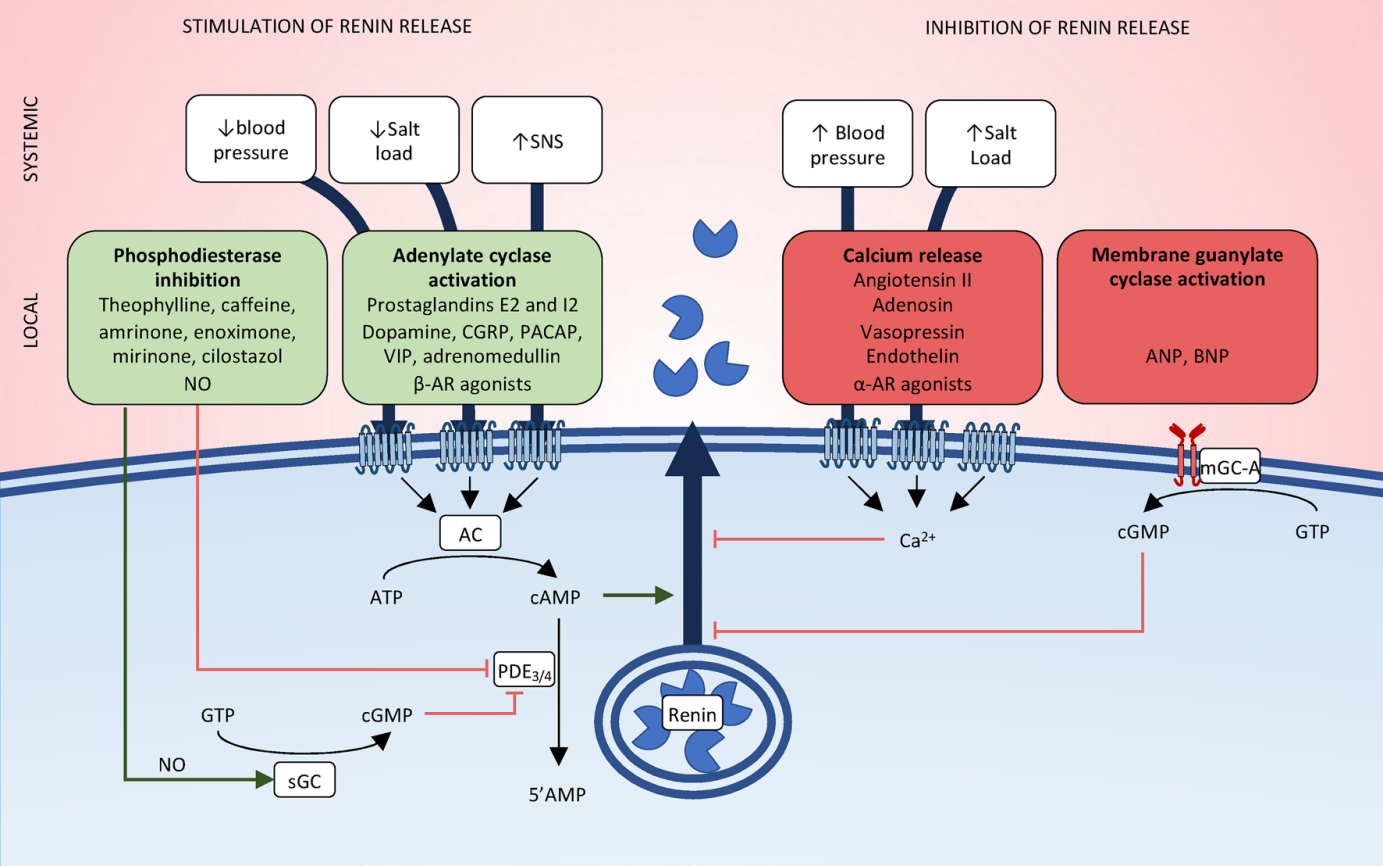
Regulation of renin secretion. Juxtaglomerular cells release renin through exocytosis in response to various stimuli. Systemic factors such as blood pressure, salt intake, and activation of the sympathetic nervous system exert their effects, in part, through the mediators described here. At the cellular level, all molecular mediators that stimulate renin release do so by elevating intracellular cAMP concentration. Conversely, any stimuli associated with intracellular calcium accumulation inhibit renin release. cGMP can either stimulate or inhibit renin secretion depending on its mode of production. Activation of sGC by NO inhibits PDE3 and thus stimulates renin release through cAMP accumulation. Conversely, activation of mGC-A by ANP or BNP inhibits renin release. *SNS* sympathetic nervous system, *NO* nitric oxide, *AC* adenylate cyclase, *mGC-A* membrane guanylate cyclase A, *sGC* soluble guanylate cyclase, *PDE3/4* phosphodiesterase 3/4, *ATP* adenosine triphosphate, *cAMP* cyclic adenosine monophosphate, *GTP* guanosine triphosphate, *cGMP* cyclic guanosine monophosphate

The renin–angiotensin–aldosterone system is a complex network of over thirty peptides, enzymes, receptors, and associated proteins (Fig. 1). The formation of all RAAS effector peptides occurs through the sequential proteolytic cleavage of angiotensinogen, also known as the RAAS proteolytic cascade. Angiotensinogen is an α_2_-glycoprotein produced by hepatocytes and cleaved by renin to generate the inactive decapeptide angiotensin I. Renin is an enzyme produced by the juxtaglomerular cells of the kidney with two modes of release: a constitutive release of an inactive or pro-renin form; and a regulated pulsatile release of active renin, influenced by various stimuli, most notably a drop in renal perfusion pressure, a low salt intake or sympathetic nervous system activation (Fig. 2) [5]. This finely tuned regulation of renin release operates within minutes to ensure a precise and timely adjustment of RAAS input and its downstream effects. A slower induction of angiotensinogen synthesis and release occurs in response to multiple stress factors such as glucocorticoids, cytokines (interleukin-1 and -6, interferon-γ, tumor necrosis factor α), estrogens, triiodothyronine and angiotensin II (via a positive feedback loop), aiming at maintaining a sufficient substrate supply during prolonged stress conditions [6]. However, it should be noted that the synthesis of angiotensinogen depends on the integrity of hepatic function [7]. Since the circulating angiotensinogen concentration is close to the Michaelis–Menten constant of renin, even discrete changes in its concentration are susceptible to affect angiotensin I generation [8].

The inactive peptide angiotensin I is subsequently cleaved into the principal RAAS effector: the octapeptide angiotensin II by the action of the angiotensin-converting enzyme (ACE). This enzyme exists in two forms: a membrane-bound form, mainly expressed by pulmonary endothelial cells, and a soluble or circulating form. Importantly, data from the literature suggest that, at least in physiological circumstances, most of the conversion activity is carried out by membrane-bound ACE, quantitatively more important [9]. Angiotensin II exerts its main effect via the angiotensin II, type 1 receptor (AT1R), mediating vasoconstriction, cell proliferation, pro-fibrotic and pro-inflammatory signals, as well as stimulating aldosterone release from the adrenal gland [10]. Additionally, AT1R stimulation on juxta-glomerular cells inhibits renin release, in a negative biofeedback mechanism [5, 10]. Another receptor, termed the angiotensin II type 2 receptor (AT2R) elicits opposite effects [10]. Angiotensin II is further cleaved into angiotensin III by aminopeptidase A and subsequently into angiotensin IV by aminopeptidase N [10]. Alternatively, angiotensin II can be directly cleaved into angiotensin IV by dipeptidyl peptidase 3 (DPP3) [11]. Angiotensin III binds to AT1R and AT2R with similar affinity as angiotensin II, thereby producing similar effects, albeit with faster clearance [12]. Angiotensin IV also exhibits low-affinity binding to angiotensin II receptors but also possess a specific receptor initially referred to as the angiotensin IV receptor (AT4R) and later identified as the insulin-regulated aminopeptidase (IRAP), a transmembrane aminopeptidase [10]. Binding of angiotensin IV competitively inhibits IRAP, thereby extending the half-life of its other substrates such as oxytocin or vasopressin [13, 14].

Alongside this well-established “classical” RAAS, a newly recognized “alternative” system has recently been brought to light. Entry into this alternative system can occur at various level. An ACE homolog called angiotensin converting enzyme 2 (ACE2) allows the formation of angiotensin-(1–9) from angiotensin I or angiotensin-(1–7) from angiotensin II [15]. Additionally, angiotensin-(1–7) can be generated by prolyl peptidases from angiotensin II or directly from angiotensin I by neprilysin (NEP) also known as neutral endopeptidase or by thimet oligopeptidase [15]. However, the respective contributions of these enzymes to angiotensin-(1–7) generation remain poorly understood. ACE catalyzes the formation of angiotensin-(1–7) from angiotensin-(1–9) and further converts it into angiotensin-(1–5). Themain effector of this “alternative” system, angiotensin-(1–7) mediates vasodilatory, natriuretic, anti-fibrotic and anti-inflammatory effects through activation of the MAS receptor [15].

Finally, more recent investigations have unveiled the existence of a third group of RAAS peptides known as alatensins. These peptides differ from the previously described ones by the presence of an alanine in the N-terminal position, rather than an arginine. Thus far, two members of the alatensin family have been identified in vivo: angiotensin A or ala^1^-angiotensin II, and alamandine or ala^1^-angiotensin-(1–7). Angiotensin A acts as a ligand for both AT1R and AT2R while alamandin exerts its effects through the MAS-related G-protein-coupled receptor D (MRGD) [16, 17]. It is conceivable that future research may uncover additional members of the alatensin family in the years to come [18].

Overall, the classical RAAS could is generally associated with vasoconstrictive, pro-fibrotic, and pro-inflammatory effects, while the alternative RAAS act as a counter-balancing system, mediating vasodilatory, anti-fibrotic, and anti-inflammatory effects. The alatensins represents an intermediate system with angiotensin A resembling angiotensin II and alamandine resembling angiotensin-(1–7). However, due to the rapid conversion of angiotensin A into alamandine, the overall impact effect of the alatensin system tends to resembles that of the alternative RAAS [16, 17]. It is important to note that this dichotomy mainly arises from observations and experiments conducted in chronic settings that may not accurately reflect acute conditions such as circulatory failure. Furthermore, the same peptide can yield different effects depending on the receptor it interacts with (*e.g.*, angiotensin II via AT1R or AT2R) suggesting that the modulation of respective receptor abundance in acute situations may act as a potential modifier of the net peptide action.

### Current understanding of the RAAS during septic shock

#### The RAAS as a compensatory mechanism during circulatory stress

There is a distinct contrast between physiology and pathology regarding RAAS dependency. In purely physiological settings, when salt intake is sufficient and the blood pressure maintenance systems are intact, RAAS integrity appears to be non-essential. For instance, under such conditions, the administration of a renin antagonist or an AT1R antagonist leads to minimal short-term hemodynamic effects in awake healthy animals and humans [19, 20]. It is worth noting that in the same conditions, an ACE inhibitor results in a significant reduction in blood pressure because ACE also catalyzes the degradation of bradykinin, a potent vasodilator [20]. Conversely, the RAAS assumes a pivotal role in stress conditions by facilitating the maintenance of blood pressure primarily through the vasoconstrictive and anti-natriuretic actions of angiotensin II, as well as angiotensin II-induced release of aldosterone, potentiating sodium reabsorption. Therefore, prior sodium depletion unmasks the hypotensive effect of AT1R antagonist in healthy humans [21]. Patients taking RAAS inhibitors are more likely to experience general anesthesia-induced hypotension [22]. In this specific context, it has been elegantly demonstrated that the major systems of blood pressure regulation, namely, the sympathetic system, the vasopressinergic system and the RAAS, are intricately interconnected and capable of compensating for the dysfunction of one another. However when all three systems are compromised, life-threatening hypotension occur [23]. The close interconnection between these three systems is further emphasized by the evidence of synergy among them. For instance, the vasoconstrictive response to angiotensin II is enhanced in presence of norepinephrine or vasopressin [24, 25].

The short-term effect of RAAS on glomerular function relies both on the modulation of renal perfusion pressure, but also on a differential action on the two vascular poles of the glomerulus. Angiotensin II predominantly induces vasoconstriction of the efferent glomerular arteriole rather than of the afferent arteriole [26]. Consequently, the loss of AT1R-dependent vasomotor tone tends to lower glomerular capillary pressure, resulting in a decrease of glomerular filtration rate [26]. In healthy animals and humans, these hemodynamic alterations typically do not translate into significant functional changes. Thus, the administration of an AT1R antagonist to healthy mice or humans does not affect glomerular filtration rate. [27, 28]. However, in a situation of circulatory stress, the use of RAAS inhibitor precipitates the reduction of glomerular filtration rate [26].

**Fig. 3:**
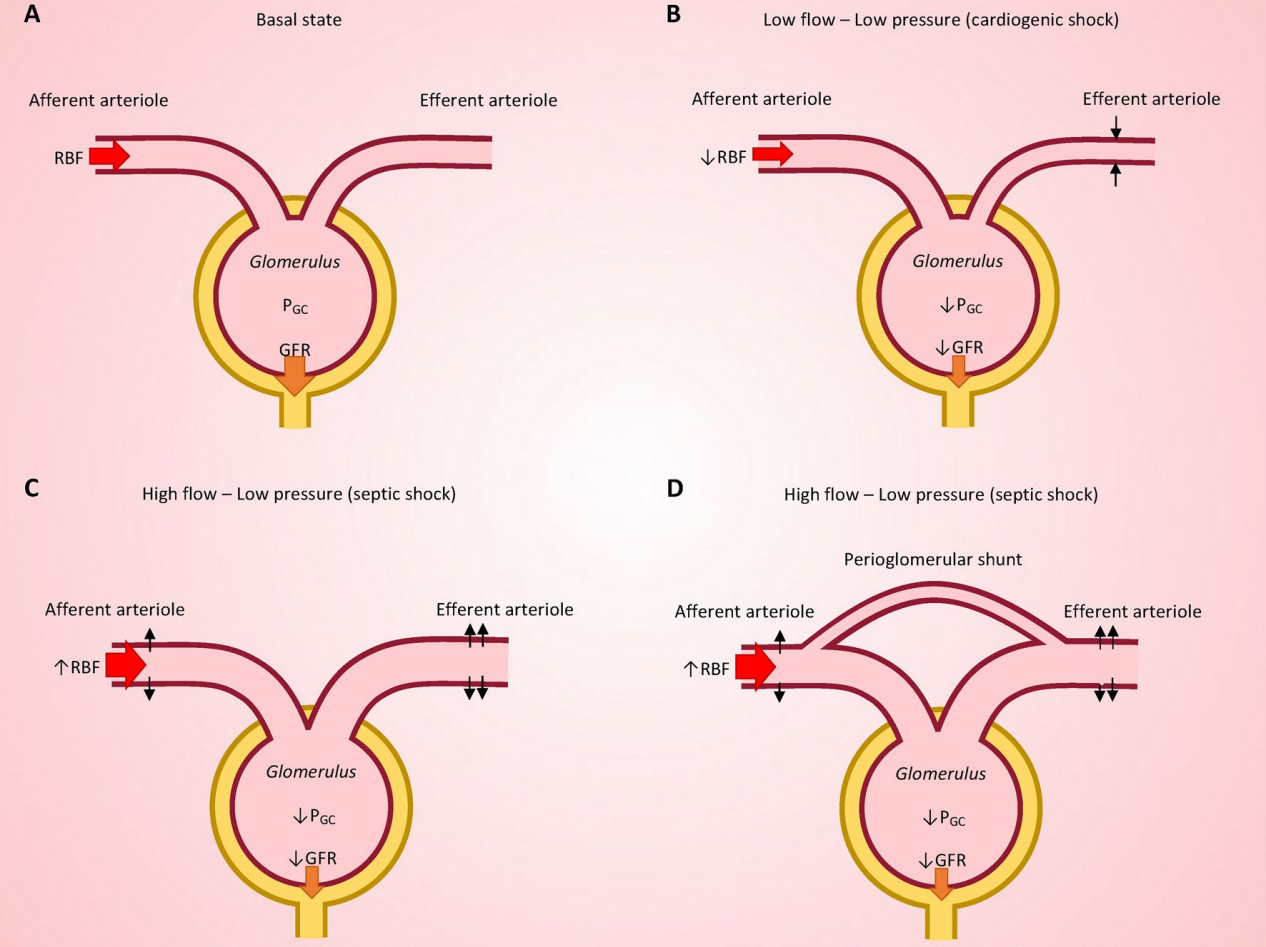
Glomerular hemodynamics in health and circulatory failure. Basal state (**A**). During circulatory failure characterized by low cardiac output (*e.g.*, cardiogenic shock), reflex myogenic vasodilation of the afferent arteriole and vasoconstriction of the efferent arteriole tend to maintain P_GC_ and therefore GFR. When these mechanisms are insufficient to maintain P_GC_, GFR falls (**B**). Conversely, during vasodilatory shock with high cardiac output (e.g., septic shock), preferential vasodilation of the efferent arteriole is sufficient to explain the fall in P_CG_ and therefore GFR decrease despite a high RBF (**C**). Additionally, reopening of peri-glomerular shunts could participate in the RBF–GFR decoupling observed during sepsis-associated acute kidney injury (**D**). *RBF* renal blood flow, *P*_*GC*_ Glomerular capillary pressure, *GFR* Glomerular filtration rate

Animal models of septic shock have provided compelling evidence regarding the crucial role of RAAS integrity in maintaining systemic and renal hemodynamic. Thus, administration of RAAS inhibitors has been associated with worse systemic hemodynamic and increased severity of acute kidney injury (AKI) in experimental septic shock [29, 30]. It is noteworthy that in the early stage of experimental and human sepsis-associated AKI, the decline in glomerular filtration rate is associated with elevated renal blood flow and reduced renovascular resistances [31]. This decoupling between flow and glomerular filtration may be explained by a preferential efferent arteriole vasodilation, resulting in the loss of post-glomerular resistance leading to decreased glomerular capillary pressure and ultimately a reduction in glomerular filtration rate (Fig. 3). While numerous factors have been implicated in the genesis of vasodilation during sepsis, the preferential involvement of the efferent arteriole suggest that angiotensin II–AT1R signaling inadequately opposes these vasodilating substances. In experimental models of septic shock, angiotensin II restores renal blood flow to control levels and is associated with improved glomerular function [30, 32]. Another hypothesis that may explain the hemodynamic profile of sepsis-associated AKI is the opening of periglomerular shunts. Although the existence of such shunts has been demonstrated in healthy animals, their role in human sepsis-associated AKI remains to be established [33]. Nevertheless, in the presence of a shunt, a decreased in efferent resistance is likely to favor the shunt, with the blood flow following the path of least resistance (Fig. 3). The observation of minimal histologic changes in early sepsis-associated AKI suggests that the aforementioned hemodynamic alterations play a significant role in renal function impairment [30, 34].

#### RAAS alterations during septic shock

Current evidence suggests that this crucial homeostatic system may falter in the context of septic shock. In critically ill patients, elevated renin concentration has been associated with lower blood pressure, a higher incidence of major adverse kidney events, and increased mortality rate and [35–38]*.* During catecholamine-resistant vasodilatory shock, mostly of septic origin, high renin concentration correlates with high circulating angiotensin I concentrations [36]*.* However, this contrasts with normal or low circulating angiotensin II concentrations [36, 39], resulting in a high angiotensin I/angiotensin II ratio which has been associated with increased mortality [39]. Importantly, these data come from a study that measured circulating angiotensin concentrations using serum to which peptidase inhibitors were added after a first freeze–thaw cycle, which could have permitted ongoing angiotensin processing during initial blood clotting step and thus represents an imperfect methodology. Despite a comparison with healthy controls whose samples were handled similarly, these observations must therefore be interpreted with caution and require confirmation.

All factors considered, the angiotensin I/angiotensin II ratio exhibits an inverse correlation with ACE activity [40]. Consequently, the constatation of a high ratio in critically ill patients has led to the attribution of the observed alterations primarily to an ACE deficiency [36, 39]. Indeed, decreased ACE activity has been found in the subgroup of septic shock patients with acute lung injury, consistent with the dominant pulmonary expression of ACE and suggesting a predominant role of sepsis-associated (pulmonary) endotheliopathy [41–43]. Additionally, impaired ACE activity could be linked to the presence of circulating endogenous ACE inhibitors formed during shock [44, 45]. Importantly, a defect of ACE activity also leads to bradykinin accumulation, further worsening vasoplegia [40].

**Fig. 4:**
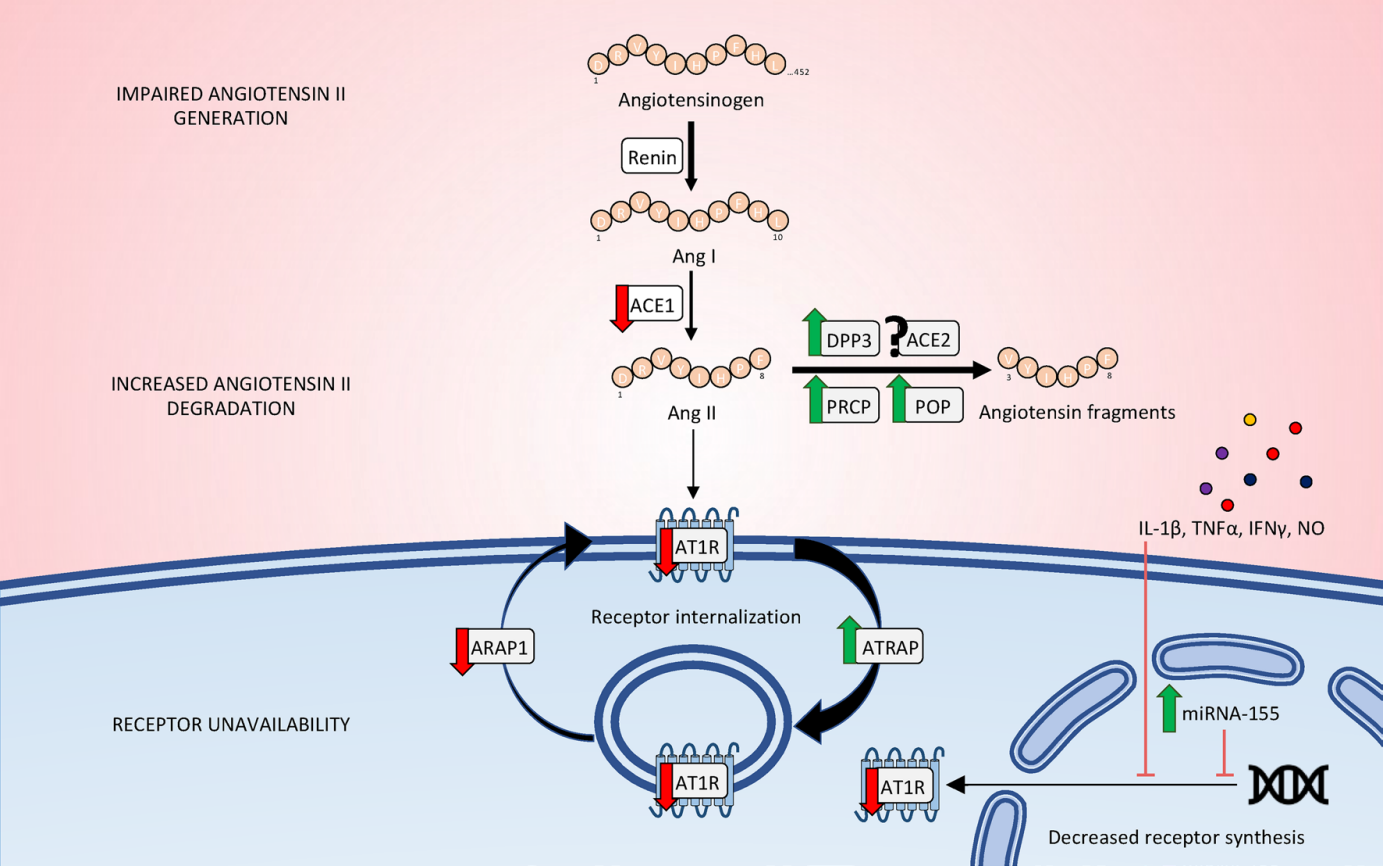
Mechanisms of impaired Ang II–AT1R signaling in septic shock. Impaired angiotensin II–AT1R signaling occur at multiple levels, including: 1. Impaired generation of angiotensin II, possibly attributable to defects in angiotensin-converting enzyme activity; 2. Enhanced degradation of angiotensin II by peptidases such as circulating DPP3, POP or PRCP; and/or 3. Unavailability of the AT1R receptor due to internalization or reduced synthesis under the influence of pro-inflammatory cytokines, NO or miRNA-155. Importantly, the angiotensin fragments that originate from angiotensin II cleavage could also mediates their own biological effects (*e.g.*, angiotensin (1–7) via ACE2, POP or PRCP; angiotensin IV via DPP3). *ACE* angiotensin-converting enzyme, *DPP3* dipeptidyl peptidase 3, *ACE2* angiotensin-converting enzyme 2, *POP* prolyl olygopeptidase, *PRCP* prolyl carboxypeptidase, *AT1R* angiotensin II, type 1 receptor, *ARAP1* AT1R-associated protein 1, *ATRAP* AT1R-associated protein. *IL-1β* interleukin-1β, *TNFα* tumor necrosis factor α, *IFN* interferon γ, *NO* nitric oxide

However, several mechanisms may be intertwined in RAAS alterations during septic shock (Fig. 4) (Table 1). In addition to a decreased generation of angiotensin II, enhanced degradation of angiotensin II by peptidases; and/or the unavailability of AT1R could also contribute to defective signaling. Early experiments already demonstrated the progressive rise of plasma angiotensinase activity during endotoxemic shock, in dogs [46]. To date, most of the evidence pertains to increased concentration and activity of circulating DPP3 [47, 48]. This enzyme hydrolyses angiotensin II but not angiotensin I, resulting in an elevated angiotensin I/angiotensin II ratio [49, 50]. Interestingly, both the baseline concentration and kinetics of circulating DPP3 are associated with outcome during sepsis and septic shock [51, 52]. Other enzymes capable of degrading angiotensin II include ACE2. Notably, ACE2 exhibits a higher affinity for angiotensin II than angiotensin I, thus suggesting that an increase in ACE2 activity could theoretically lead to an elevated angiotensin I/angiotensin II ratio [53]. Neverthelessthe role of ACE2 in increased angiotensin II degradation during septic shock remain to be determined. Other angiotensin II-degrading enzymes may also be implicated such as prolyl oligopeptidase or prolyl carboxypeptidase [54]. Additionally, it is worth noting that NEP cleaves angiotensin II into angiotensin-(1–4) and angiotensin-(5–8) and could theoretically by-pass ACE by directly generating angiotensin-(1–7) from angiotensin I. Although the circulating concentration of NEP is increased in critically ill patients [55], such increase is not associated with prognosis, potentially because concentration and activity are dissociated during septic shock, likely due to endogenous inhibitors [56, 57].

**Table 1:** Variations of circulating and tissue renin–angiotensin aldosterone-system components during experimental or human sepsis/septic shock

References	Species	Clinical condition or experimental model	Main findings
[36]	Human	Vasodilatory shock^a^	↑ Circulating renin concentration
[39]	Human	Vasodilatory shock^a^	↑ Circulating angiotensin I concentration = Circulating angiotensin II concentration
[41]	Human	Septic ARDS	↓Circulating ACE activity
[42]	Human	Septic ARDS	↓Lung ACE expression
[91]	Monkey	*E. coli* infusion	↓Circulating ACE activity
[45]	Human	Pediatric septic shock	↑ Circulating ACE concentration ↓ Circulating ACE activity
[46]	Dog	LPS	↑ Circulating angiotensinase activity
[47]	Human	Sepsis and septic shock	↑ Circulating DPP3 concentration and activity
[48]	Rat	Septic shock	↑ Circulating DPP3 activity
[54]	Human	Septic shock	↑ Circulating POP activity ↑ Circulating PRCP activity
[55]	Human	Sepsis and septic shock	↑ Circulating NEP concentration
[56]	Human	Septic shock	↓ Circulating NEP activity
[92]	Rat	CLP	↑ then ↓ Circulating NEP activity ↓ Tissue NEP expression and activity
[61]	Mouse	LPS	↓ Tissue ARAP1 expression
[62]	Rat	LPS	↑ Circulating renin activity ↑ Circulating angiotensin II concentration = Circulating aldosterone concentration ↓ Tissue AT1R expression
[88]	Mouse	CLP	↑ Neutrophils and inflammatory monocytes AT1R expression ↓ Neutrophils and inflammatory monocytes ACE expression
[30]	Mouse	CLP	↓ kidney AT1R expression
	Human	Sepsis/septic shock	↓ kidney AT1R expression

To ensure clarity, this table exclusively presents studies that incorporate comparisons with an appropriate control group (healthy subjects, critically ill patients without infection or pre-morbid animals) and/or utilize assays with well-established reference ranges

*LPS* lipopolysaccharide, *CLP* cecal ligation and puncture, *ARDS* acute respiratory distress syndrome, *ACE* angiotensin-converting enzyme, *DPP3* dipeptidyl peptidase 3, *POP* prolyl oligopeptidase, *PRCP* Prolyl carboxypeptidase, *NEP* neprilysin, *AT1R* Angiotensin II, type 1 receptor, *ARAP1* AT1R-Associated Protein 1

^a^Including more than 80% septic shock patients

Finally, a reduced sensitivity to angiotensin II in septic shock models and patients compared to their healthy counterparts suggests a downstream defect. This impaired signaling could arise from AT1R unavailability, which may result from AT1R internalization or reduced synthesis. The membrane localization of AT1R is known to be modulated by two associated proteins: the AT1R associated protein (ATRAP), which enhances AT1R internalization, and the AT1R associated protein 1 (ARAP1), promoting the recycling of AT1R to the membrane [58, 59]. Interestingly, a single nucleotide polymorphism of *ATRAP* has been associated with increased ATRAP protein expression in vitro and with increased vasoplegia in patients, as demonstrated by decreased mean arterial pressure during sepsis and post-cardiac surgery [60]. These findings are further linked to increased mortality of patients presenting such polymorphism during septic shock [60]. Conversely, expression of ARAP1 is downregulated during experimental endotoxemia or after in vitro exposition to pro-inflammatory cytokines [61]. Furthermore, experimental endotoxemia in *Arap1* knockout mice suggests that the downregulation of ARAP1 expression during sepsis contributes to the development of hypotension by reducing vascular sensitivity to angiotensin II [61]. Additionally, nitric oxide and pro-inflammatory cytokines may cooperate to decrease *AT1R* gene transcription [62, 63]. Micro-RNA-155, which is overexpressed in both animal and human sepsis, negatively regulates *AT1R* transcription, ultimately leading to decreased vasoconstrictive responses to angiotensin II [64].

Importantly, the growing utilization of RAAS inhibitors exposes patients to iatrogenic causes of defective AT1R signaling. For instance, the use of renin inhibitor or ACE inhibitors is likely to contribute to an insufficient generation of angiotensin II. Conversely, the use of an angiotensin receptor blocker reduces sensitivity to angiotensin II [65]. It is worth noting that some of these medications have a prolonged half-life and are eliminated through the renal route, exposing patients to long-lasting alterations.

The aforementioned alterations can occur either in isolation or in combination, leading to an uncoupling between RAAS input (renin release) and output (AT1R stimulation). Intriguingly, evidence of such uncoupling could be found in situations of hyperreninemic hypoaldosteronism described several years ago in a subset of critically ill patients while the underlying mechanism remain elusive. These patients, despite consistently elevated plasma renin activity, exhibited abnormally normal or even low plasma aldosterone concentration. Notably, this biochemical profile was more frequently observed in septic shock patients and was associated with higher incidence of AKI as well as lower survival rates [66–68].

### RAAS-targeted therapy

The only RAAS targeted therapy that has advanced to clinical stages in shock is angiotensin II. In animal models of septic shock, vasopressor support with angiotensin II has been associated with similar systemic hemodynamics compared to norepinephrine [29, 69]. The use of angiotensin II in humans was initially limited to bovine angiotensin II in cases of refractory shock [70, 71]. The development of synthetic human angiotensin II allowed larger-scale evaluation.

The randomized controlled trial ATHOS-3 investigated the efficacy of angiotensin II on top of standard of care in 344 patients with catecholamine-refractory vasodilatory shock, predominantly of septic origin [4]. The primary end point, response with respect to mean arterial pressure at hour 3 after the start of infusion (a response was defined as an increase from baseline of at least 10 mm Hg or an increase to at least 75 mm Hg, without an increase in the dose of background vasopressors), was reached more often in patients randomized to angiotensin II, compared to those receiving placebo (69.9% vs 23.4%, p < 0.001). However, there were no significant difference in secondary outcomes such as the mean change in SOFA score at hour 48, or mortality at day 7 or 28. *Post-hoc* analyses suggested that in patients with AKI requiring renal replacement therapy at study drug initiation, the rate of renal replacement therapy liberation and 28-day survival were greater in the angiotensin II group compared to the placebo group [72]. Despite these interesting and biologically plausible findings, the *post-hoc* nature of the analysis and the absence of predefined criteria for initiating and discontinuing renal replacement therapy prevent definitive conclusions. Additionally, in patients with baseline renin concentration above the median of ATHOS-3 population, angiotensin II was associated with a significant reduction in 28-day mortality, compared to placebo (50.9% vs 69.9%, p = 0.012), suggesting the potential use of renin as an enrichment biomarker in future trials. At last, in the subgroup of ATHOS-3 patients with acute respiratory distress syndrome, randomization to the angiotensin II arm was associated with improved oxygenation [73]. Despite these encouraging findings, it remains to be prospectively demonstrated that angiotensin II is associated with improved patients-centered outcomes such as survival. Additionally, several questions remain to be answered.

First, it is unclear whether angiotensin II should be administered in all patients with vasodilatory shock. Noticeably, the vasopressor response to angiotensin II is not constant. In ATHOS-3, 30% of patients randomized to angiotensin II did not have a blood pressure response at hour-3 despite the use of large doses (up to 200 ng/kg/min) [4]. Conversely, a hyper-response phenomenon has also been described [74–76]. This diversity of response profile could mirror the diversity of RAAS alterations mechanisms described earlier. According to this hypothesis, patients with an isolated angiotensin II generation defect would be likely to be hyper-responsive to exogenous angiotensin II. Conversely, high doses might not be sufficient in patients with increased degradation of angiotensin II and/or AT1R unavailability. In these latter patients, alternative or complementary strategies might be necessary. Second, in ATHOS-3, 80.7% of patients had septic shock. Whether angiotensin II is of benefit in non-septic vasodilatory shock remain to be investigated. The molecular mechanisms underlying RAAS alterations have been primarily described in septic contexts. However, similar alterations might be encountered in non-septic vasodilatory shock. Indeed, elevated renin is associated with prolonged need of vasopressors and the occurrence of acute kidney injury after cardiopulmonary bypass surgery [77]. Nevertheless, the benefit-risk balance of exogenous angiotensin II administration in non-septic situations may differ. Patients with recent cardiac injuries, may raise concerns, and dedicated studies are needed to assess the appropriateness of angiotensin II support in these cases. Third, in ATHOS-3, more than two thirds of subjects were receiving two or more vasopressors prior to study drug administration. Additional research is required to determine whether angiotensin II should be administered as the primary vasopressor or as a secondary or tertiary option. Evaluating the potential benefits of a primary balanced strategy combining norepinephrine, vasopressin, and angiotensin II should also be considered [78].

Finally, the adverse event profile needs further examination. While the rates of adverse events of special interest were similar between the angiotensin II and placebo groups in ATHOS-3, there was a higher incidence of combined arterial and venous thromboembolic events in patients receiving angiotensin II [4, 79]. This observation aligns with the known pro-thrombotic properties of angiotensin II and has led to recommendations for thromboembolism prophylaxis in patients treated with angiotensin II [80].

### Future directions and recommendations for RAAS research in experimental models and critically ill patients

We have attempted to synthesize the available translational evidence regarding RAAS alterations during septic shock, their consequences, and potential therapeutic strategies. Nevertheless, further investigation is warranted to gain a comprehensive understanding of RAAS alterations during septic shock. Notably, investigation of alternative RAAS components such as ACE2 and angiotensin-(1–7) appear critical for unraveling the complex contribution of RAAS alterations to circulatory failure. Additionally, exploring the temporal aspects of RAAS alterations throughout the course of disease is essential to inform the selection of optimal therapeutic approaches at different stages of septic shock. Furthermore, a pressing need exists to identify distinct patient phenotypes based on RAAS alterations and translate this understanding into personalized interventions. To achieve a comprehensive understanding of the RAAS in septic shock, rigorous research methodologies and adequate experimental models are indispensable. In this regard, several important points should be highlighted.

Renin measurement alone is insufficient to draw conclusion about the pathophysiological role of the RAAS in pathological situation. Indeed, high renin can result from appropriate feedback to an impaired AT1R signaling or from inappropriate activation of the RAAS leading to more inflammation, oxidative stress and intra-renal vasoconstriction [81]. When the aim is to investigate the mechanistic role of the RAAS in a pathological condition, it appears preferable to couple renin and RAAS peptides measurements and to assess their downstream clinical (*e.g.,* systemic and renal hemodynamic) and biological (*e.g.,* aldosterone secretion) effects. Importantly, when considering the specific role of renin, it is advisable to prioritize an assay measuring active renin concentration or a standardized activity assay with the addition of exogenous angiotensinogen, over the historical “plasma renin activity”. Indeed, plasma renin activity reflects the overall RAAS input and rely not only on renin concentration but also on angiotensinogen concentration in the sample [82]. To prevent falsely elevated results caused by the cryoactivation of pro-renin that occurs within a temperature range of 2 to 8 °C, samples should be frozen and thawed as quickly as possible, and the assay performed at room temperature [83].

Measuring RAAS peptides is challenging and necessitates validated and reproducible methodology. Such measurements face multiple obstacles including short half-life, low concentrations, and high sequence similarity of RAAS peptides as well as *ex-vivo* generation from circulating angiotensinogen and renin. These pitfalls might be adequately addressed by the use of plasma samples stabilized by peptidases inhibitors upon blood collection and by the use of modern measurements methods such as liquid chromatography coupled with immunoassays or mass spectrometry or [82, 84]. Due to the prolonged clotting time required, the use of serum samples should be avoided. A precise description of the methodology used should always be reported.

To gain a better understanding of the precise mechanism underlying a potential benefit of RAAS modulation, it is desirable to study not only the initial RAAS picture during shock, but also how a specific intervention modifies the system. For instance, the benefits associated with angiotensin II administration may extend beyond the directly observable AT1R-mediated hemodynamic effects, involving additional mechanisms. Negative feedback on renin release could reduce pro-inflammatory effects mediated by renin via the (pro)renin receptor [85–87]. Other AT1R-mediated immune effects could participate in pathogen clearance during sepsis [88]. Additionally, exogenous angiotensin II could exert beneficial effects directly through the AT2R receptor or after in vivo conversion into alternative RAAS peptides or alatensins [89].

The translation of observations obtained in animal studies to humans must be done cautiously, given numerous inter-species variations [90]. Therefore, whenever possible, these observations should be validated in several species and, at best, in humans.

From a clinical perspective, the short-term benefit of RAAS modulation should not overshadow the potential long-term side effects associated with the well-established AT1R-mediated pro-inflammatory and pro-fibrotic effects. Therefore, especially when planning human trials, long-term follow-up with vigilant monitoring for adverse cardiovascular and/or renal events is advisable.

## Conclusion

While the RAAS is crucial to cope with circulatory stress, alterations of this homeostatic system have been recently demonstrated during septic shock, ultimately leading to impaired angiotensin II signaling. These observations open a new field of research and hopefully new therapeutic avenues susceptible to improve patients’ prognosis. In the next future, a better understanding of RAAS alterations should help to decipher patients’ phenotypes and translate into targeted interventions.

## Data Availability

Not applicable.

## Abbreviations

RAAS: Renin–Angiotensin–Aldosterone System
ACE: Angiotensin-Converting Enzyme
AT1R: Angiotensin II, Type 1 Receptor
AT2R: Angiotensin II, Type 2 Receptor
DPP3: DiPeptidyl Peptidase 3
AT4R: Angiotensin IV receptor
IRAP: Insulin-Regulated AminoPeptidase
ACE2: Angiotensin-Converting Enzyme
NEP: Neprilysin/neutral endopeptidase
MRGD: MAS-related G-protein-coupled D receptor
AKI: Acute kidney injury
ATRAP: AT1R-Associated Protein
ARAP1: AT1R-Associated Protein 1
